# Identification of a Prognostic Clinical Score for Patients With Recurrent or Metastatic Squamous Cell Carcinoma of the Head and Neck Treated With Systemic Therapy Including Cetuximab

**DOI:** 10.3389/fonc.2021.635096

**Published:** 2021-05-13

**Authors:** Michael Pogorzelski, Thomas Hilser, Saskia C. Ting, Benjamin Kansy, Thomas C. Gauler, Martin Stuschke, Kurt W. Schmid, Stephan Lang, Viktor Grünwald, Martin Schuler, Stefan Kasper

**Affiliations:** ^1^ Department of Medical Oncology, West German Cancer Center, University Hospital Essen, Essen, Germany; ^2^ Institute of Pathology, West German Cancer Center, University Hospital Essen, Essen, Germany; ^3^ Department of Otorhinolaryngology, West German Cancer Center, University Hospital Essen, Essen, Germany; ^4^ Department of Radiation Oncology, West German Cancer Center, University Hospital Essen, Essen, Germany; ^5^ German Cancer Consortium (DKTK), Essen, Partner Site University Hospital Essen, Essen, Germany; ^6^ Department of Urology, West German Cancer Center, University Hospital Essen, Essen, Germany

**Keywords:** cetuximab, head and neck cancer, prognostic markers, predictive markers, systemic treatment, resistance mechanism, prognostic score

## Abstract

Cetuximab-based chemoimmunotherapy has been the standard of care for recurrent or metastatic squamous cell carcinoma of the head and neck (r/m SCCHN) for more than a decade. To date, no predictive or prognostic biomarkers have been established to further guide the systemic treatment with cetuximab-based chemoimmunotherapy in r/m SCCHN. Against this background, we retrospectively analyzed clinical and blood-based parameters from 218 r/m SCCHN patients treated with chemoimmunotherapy including cetuximab. Multivariate Cox-regression models were used to assess their prognostic or predictive value. Eastern Co-operative Oncology Group (ECOG) performance status (≥2), older age (≥61.8 years), anemia (hemoglobin <11.80), and increased neutrophil-to-lymphocyte ratio (NLR ≥5.73) were independently and strongly associated with inferior overall survival (OS). To group patients according to risk profiles we established a prognostic clinical score (PCS) that can easily be used in clinical practice. The PCS stratified the cohort into low, intermediate, poor or very poor risk subgroups with median OS times of 23.4, 12.1, 7.5, and 4.0 months, respectively. Patients with low risk PCS had a prolonged progression-free survival (PFS) and increased overall response rate (ORR) under first-line cetuximab-based therapy. Interestingly, only patients with low and intermediate risk benefitted from the more intensive first-line cisplatin/cetuximab combination compared to carboplatin/cetuximab therapy, whereas the intensity of first-line treatment had no impact in the poor and very poor risk subgroups. Following external validation, particularly in the context of newly established first-line options, the PCS may guide clinical decision making and serve for stratification of patients with r/m SCCHN in future clinical trials.

## Introduction

Squamous cell carcinoma of the head and neck (SCCHN) is the sixth most common cancer worldwide with more than 600,000 death annually. Platinum-based systemic chemotherapy in combination with the monoclonal antibody cetuximab, targeting the epidermal growth factor receptor (EGFR) has been the standard of care for recurrent or metastatic (r/m) SCCHN for over a decade (1, 2). Recently, the immune checkpoint inhibitors (CPIs) nivolumab and pembrolizumab have changed the therapeutic landscape of patients with r/m SCCHN. Nivolumab is considered as therapeutic standard after failure of platinum-based therapy based on the results of the phase III Checkmate-141 trial (3). In addition, pembrolizumab has become the new standard in the first-line setting in combination with platinum/fluorouracil chemotherapy or as monotherapy for patients with programmed cell death ligand 1 (PD-L1) positive tumors based on the data from the phase III Keynote-048 trial (4). However, response rate and progression-free survival upon CPI therapy in patients with r/m SCCHN are still disappointing and most patients will receive cetuximab and/or cytotoxic drugs in further lines (5). So far, no prognostic or predictive biomarkers have been established to stratify the systemic treatment of patients with r/m SCCHN and to identify patients with the highest likelihood of response to cetuximab (6). Ideal biomarkers are easily accessible, cost-effective and can reproducibly discriminate patients with different risks. Clinical and laboratory routine parameters could help to establish nomograms or prognostic clinical scores (PCS) to identify patients with higher chance of response or higher risk of progression upon different therapies. For example, the Eastern Co-operative Oncology Group performance status (ECOG) or the age has been correlated with a poor prognosis in a variety of cancer entities (7–9). In addition, systemic inflammatory response (SIR) parameters like the C-reactive protein (CRP), the neutrophil–lymphocyte ratio (NLR), anemia and others have been correlated with the risk of recurrence and overall survival in curative treated malignancies (9–11). The prognostic impact of these blood based SIR markers was also validated in patient receiving palliative systemic chemo- and immunotherapy (12, 13).

Against this background, we tested whether clinical and blood-based parameters had prognostic and predictive value in patients with r/m SCCHN treated with cetuximab-based therapy. The identified prognostic markers were combined to develop a new prognostic clinical score (PCS), which stratifies patients into four different risk groups. In addition, the PCS characterizes a group of patients with a higher likelihood for response and prolonged survival due to cisplatin/cetuximab-based therapy.

## Patients and Methods

### Study Design

Patients with r/m SCCHN treated with cetuximab-based systemic therapy between October 2006 and February 2018 at the West German Cancer Center (WTZ), University Hospital Essen, were retrospectively enrolled into this study. Follow-up was routinely assessed and documented in the electronic health record (EHR). Clinical and routinely assessed blood-based parameters, administered therapies and radiological response parameters were also retrieved from the EHR. The data cut-off for follow-up was October 1, 2019. Statistical and correlative analyses were performed using SPSS Statistics (V26, IBM, Armonk, NY, USA) and MS Excel 2010 (VS 14.0, Microsoft, Richmond, WA, USA). The study was approved by the local Ethics Committee of the Medical Faculty of the University Duisburg-Essen (Az 13-5486-BO).

### Assessments and Statistical Analysis

Routine staging procedures included computed tomography (CT) scan and/or magnetic resonance imaging (MRI) of the head and neck, a thoracic CT and a CT of the abdomen and/or abdominal ultrasound. Initial staging was performed according to the seventh edition (2010) of the International Union Against Cancer (UICC) TNM classification. In the recurrent or metastatic setting patients were radiologically examined routinely every 6–8 weeks during the palliative therapy according to the institutional guidelines of the West German Cancer Center. Overall response rate was evaluated according to the Response Evaluation Criteria in Solid Tumors 1.1 (RECIST 1.1) (14, 15). Response assessment was feasible if at least one baseline CT or MRI and one follow-up imaging was available. Overall survival was defined as time from first administration of systemic treatment to death from any cause. Patients were censored at the time of last follow-up, if time point of death was not evaluable. Progression-free survival (PFS) was defined as time from start of therapy to date of radiologic or clinical progression or death. For survival analyses Kaplan–Meier method and log-rank test were used. For univariate and multivariate analyses of blood-based and clinical parameters a Cox proportional-hazard model with Hazard ratios (HR) and 95% confidence intervals (CI) were applied. Differences in overall response were dissected using the chi-square test. Overall, *P*-values ≤0.05 were regarded statistically significant.

### Clinical- and Blood-Based Parameters

Based on a review of the literature, the following five blood-based parameters including markers of systemic-inflammatory response (SIR) were assessed: C-reactive protein (CRP), neutrophil–lymphocyte ratio (NLR), lymphocyte–monocyte ratio (LMR), platelet–lymphocyte ratio (PLR) and anemia (hemoglobin—HB). The three analyzed clinical markers included age, ECOG performance status and the extent of disease (local disease only *vs* metastatic disease). All parameters were assessed up to five days before the administration of the first palliative chemotherapy. For explorative analyses, we choose the median as cut-off for all blood-based parameters. We also used the median as cut-off for the clinical parameter age. For ECOG we distinguished between ECOG PS 0–1 and ≥2 and for the extent of disease we differentiated between patients with local disease only or metastatic disease.

## Results

### Patients’ Characteristics

A total of 218 patients with r/m SCCHN treated with cetuximab-based chemoimmunotherapy were enrolled into this study. Baseline clinical characteristics are listed in **Table 1**. The median age was 61.8 years (range 26.9–90.2). Notably, 39 patients (17.9%) were older than 70 years at start of the palliative therapy and 69 patients (31.6%) had an ECOG performance status of 2 or higher. Most patients were male (*N* = 173, 79.4%). One patient had an undifferentiated carcinoma (0.5%), all other had a keratinizing (*N* = 145, 66.5%) or non-keratinizing squamous cell carcinoma (*N* = 72, 33.0%). The main primary tumor site was the oropharynx (*N* = 95, 43.6%) followed by larynx (*N* = 40, 18.3%), hypopharynx (*N* = 37, 17.0%) and oral cavity (*N* = 32, 14.7%), respectively. In total, 42 patients (19.3%) presented with metastases, mostly in the lung at time point of initial diagnosis. At start of palliative therapy, 114 patients (52.3%) had distant metastasis and 104 (47.7%) had local recurrence only.

**Table 1:** Baseline clinical characteristics (*N* = 218).

	%	*N*
Median age	61.8 (range 26.9–90.2)	
age >65 years	38.5	84
age >70 years	17.9	39
age >75 years	7.3	16
Gender Female	20.6	45
ECOG		
ECOG 0–1	61.5	134
ECOG ≥2	31.6	69
n.d.	6.9	15
Primary tumor site		
nasopharynx	5.0	11
oral cavity	14.7	32
oropharynx	43.6	95
* p16 positive*	*15.8*	*15*
* p16 negative*	*28.4*	*27*
* n.d.*	*55.7*	*53*
hypopharynx	17.0	37
larynx	18.3	40
Carcinoma of unknown primary site with cervical lymph node	1.4	3
TNM-status at primary diagnosis (7^th^ edition)		
T-status		
T1	12.0	26
T2	21.1	46
T3	23.9	52
T4	37.2	81
n.d.	6.0	13
N-status*		
N0	24.8	54
N1	11.5	25
N2	55.9	122
N3	3.7	8
n.d.	4.1	9
M-status		
M0	77.5	169
M1	19.3	42
n.d.	3.2	7
Primary histology		
keratinizing SCC	66.5	145
non-keratinizing SCC	33.0	72
undifferentiated carcinoma	0.5	1
Grading		
G1	2.3	5
G2	61.9	135
G3	23.9	52
G4	1.4	3
n.d.	10.6	23
Local disease only	47.7	104
Metastatic disease	52.3	114
n.d.	7.2	11

*7^th^ edition (independent of HPV status); n.d., not documented; SCC, squamous cell carcinoma.

### Therapy

The primary tumor was resected in 126 patients (57.8%) at time point of initial diagnosis, 89 patients of these (70.6%) received an adjuvant or additive radio- or radio-chemotherapy (**Table 2**). In total, 63 patients (28.9%) underwent definitive radio-chemotherapy. In the recurrent or metastatic setting all patients were treated with a palliative systemic therapy. The majority of patients (*N* = 114, 52.3%) received second-line systemic therapy and a substantial number of patients (*N* = 66, 30.3%) underwent at least third- or further line therapies. Cetuximab-based therapy was administered in 172 patients (78.9%) in the first-line setting and in 42 patients (19.3%) in second-line. Nearly all patients (*N* = 199, 91.3%) received platinum-based therapies (cisplatin or carboplatin) in combination with 5-fluorouracil (*N* = 182, 83.4%) or a taxane (*N* = 17, 7.8%) in first-line.

**Table 2:** Therapy (*N* = 218).

	%	*N*
Surgical resection of primary tumor	57.8	126
adjuvant radio–or radio-chemotherapy	70.6	89
Definitive radiochemotherapy	28.9	63
n.d.	3.2	7
Median lines of palliative therapy	2 (range 1–6)	
Sequential therapy lines		
first line	100	218
second line	52.3	114
third line	30.3	66
fourth line	11.0	24
fifth line	3.2	7
sixth line	0.9	2
Cetuximab		
cetuximab in first line	78.9	172
cetuximab in second line	19.3	42
cetuximab in third line	1.8	4
First line		218
platinum/5-fluorouracil-based combination therapy	83.4	182
taxan-based combination therapy	7.8	17
5-fluorouracil + cetuximab	0.9	2
monotherapy	7.8	17
Second line		114
combination therapy	36.8	42
monotherapy	63.2	72
Third line		66
combination therapy	24.2	16
monotherapy	75.7	50

n.d., not documented.

### Outcome

**Figure 1:**
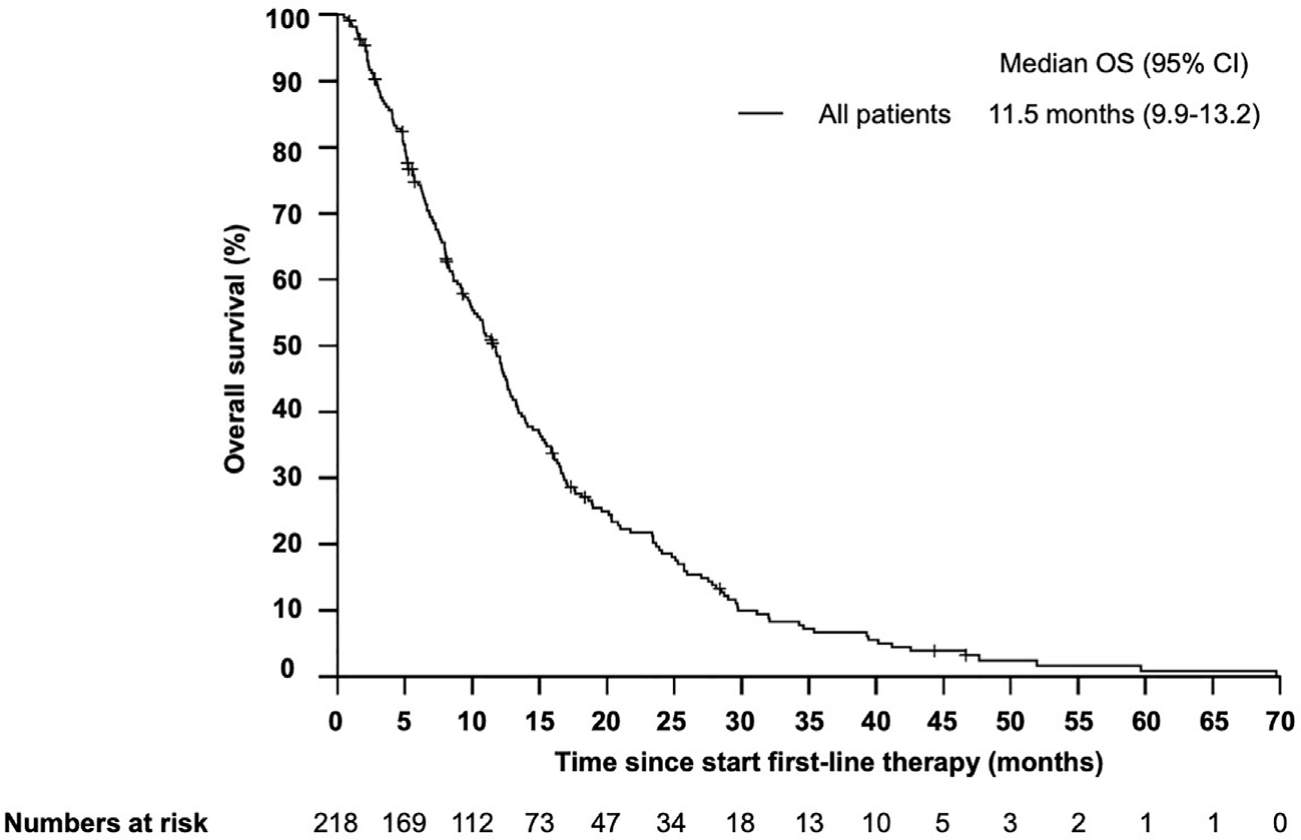
Kaplan–Meier plot for overall survival (OS) in patients with r/m SCCHN from start of first-line therapy. The median overall survival was 11.5 months (95% CI 9.9–13.2 months).

**Figure 2:**
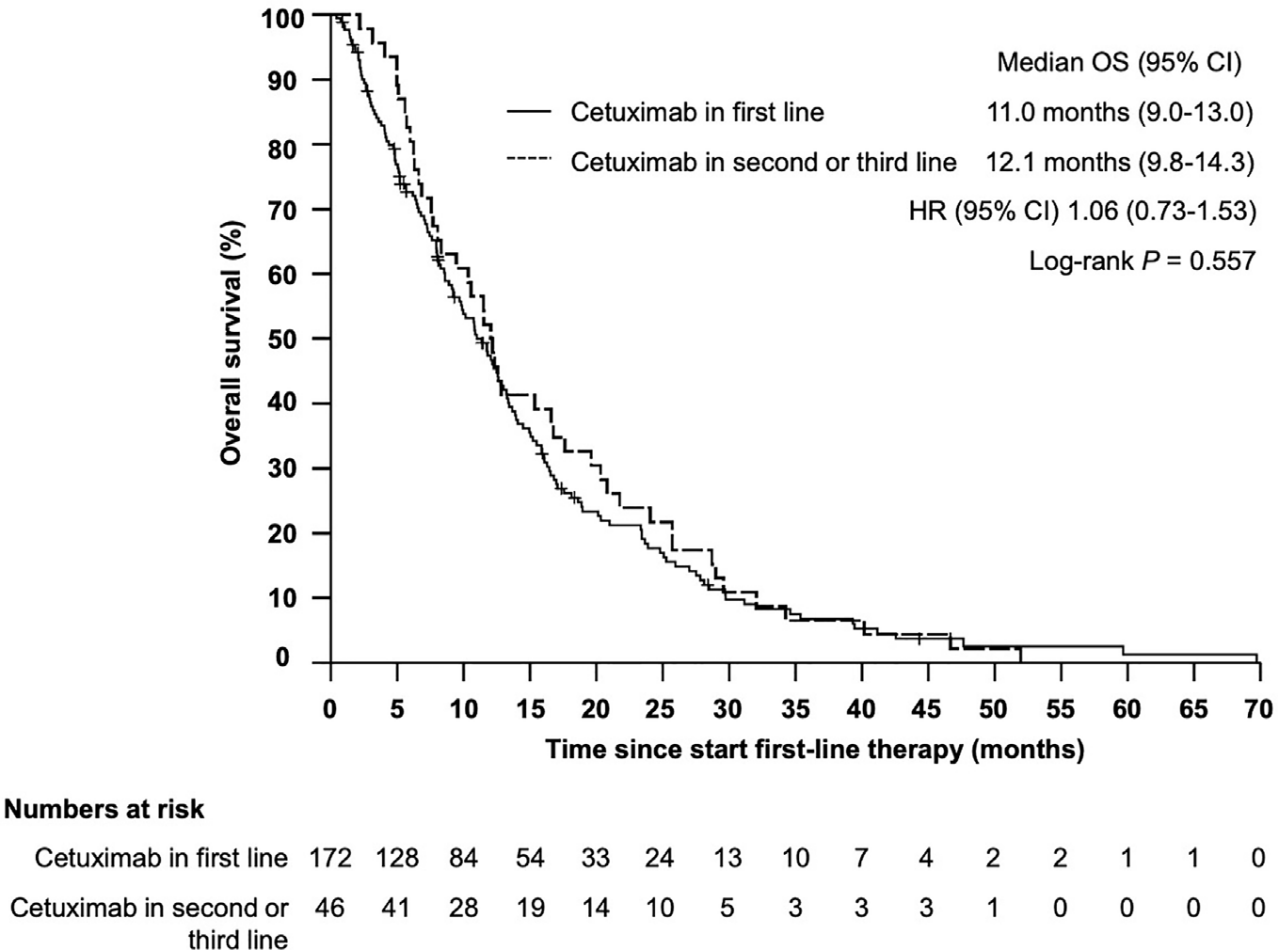
Kaplan–Meier plot for overall survival (OS) from start of first-line therapy for patients receiving cetuximab in first *vs* cetuximab in second or third line. The median overall survival was 11.0 months (95% CI 9.0–13.0 months) *vs* 12.1 months (95% CI 9.8–14.3), *P* = 0.557.

Median overall survival (OS) from start of first-line therapy was 11.5 month (95% CI 9.9–13.2) (**Figure 1**). Interestingly, there was no statistically significant difference in OS for patients who received cetuximab in first line compared with those patients who received cetuximab in second or third line (11.0 months *vs* 12.1 month, HR 1.06, 95% CI 0.73–1.53, *P* = 0.557) (**Figure 2**). In total 199 patients (91.3%) were evaluable for response according to RECIST 1.1. Overall response rate (ORR) was 27.1% under first-line therapy, including five patients (2.5%) with complete remission (CR) and the disease control rate (DCR) was 68.3% (**Table 3**). The first-line progression-free survival (PFS) was 2.6 month (95% CI 2.3–2.9).

**Table 3:** Efficacy of palliative first-line therapy (*N* = 199).

	%	*N*
ORR to first-line therapy (RECIST 1.1)		199
CR	2.5	5
PR	24.6	49
SD	41.2	82
PD	31.7	63
ORR (CR+PR)	27.1	54
DCR (CR+PR+SD)	68.3	136

ORR, overall response rate; CR, complete remission; PR, partial remission; SD, stable disease; PD, progressive disease; DCR, disease control rate.

### Explorative Prognostic Clinical- and Blood-Based Parameters

Based on a review of the literature, eight parameters were evaluated as potential prognostic markers of survival outcomes: the blood-based parameters C-reactive protein (CRP), neutrophil–lymphocyte ratio (NLR), lymphocyte–monocyte ratio (LMR), platelet–lymphocyte ratio (PLR) and anemia (hemoglobin—HB) and the clinical factors age, ECOG performance status and the extent of disease (local disease only *vs* metastatic disease). For all blood-based parameters, the median was chosen as cut-off, as listed in **Table 4**.

**Table 4:** Laboratory characteristics at start of palliative therapy for r/m SCCHN.

	Median (range)	*N*
White blood count (/nl)	7.71 (1.37–28.84)	210
Neutrophils (/nl)	5.64 (0.78–27.68)	193
Lymphocytes (/nl)	1.00 (0.17–3.74)	192
Monocytes (/nl)	0.65 (0.01–8.40)	193
Hemoglobin (g/dl)	11.80 (7.4–16.3)	210
Platelets (/nl)	293.0 (84–640)	210
CRP (mg/l)	2.30 (0.0–42.90)	205
NLR	5.73 (0.71–65.35)	192
LMR	1.55 (0.13–32.0)	192
PLR	312.13 (67.22–2305.26)	192

NLR, neutrophil–lymphocyte ratio; LMR, lymphocyte–monocyte ratio; PLR, platelet–lymphocyte ratio.

**Figure 3:**
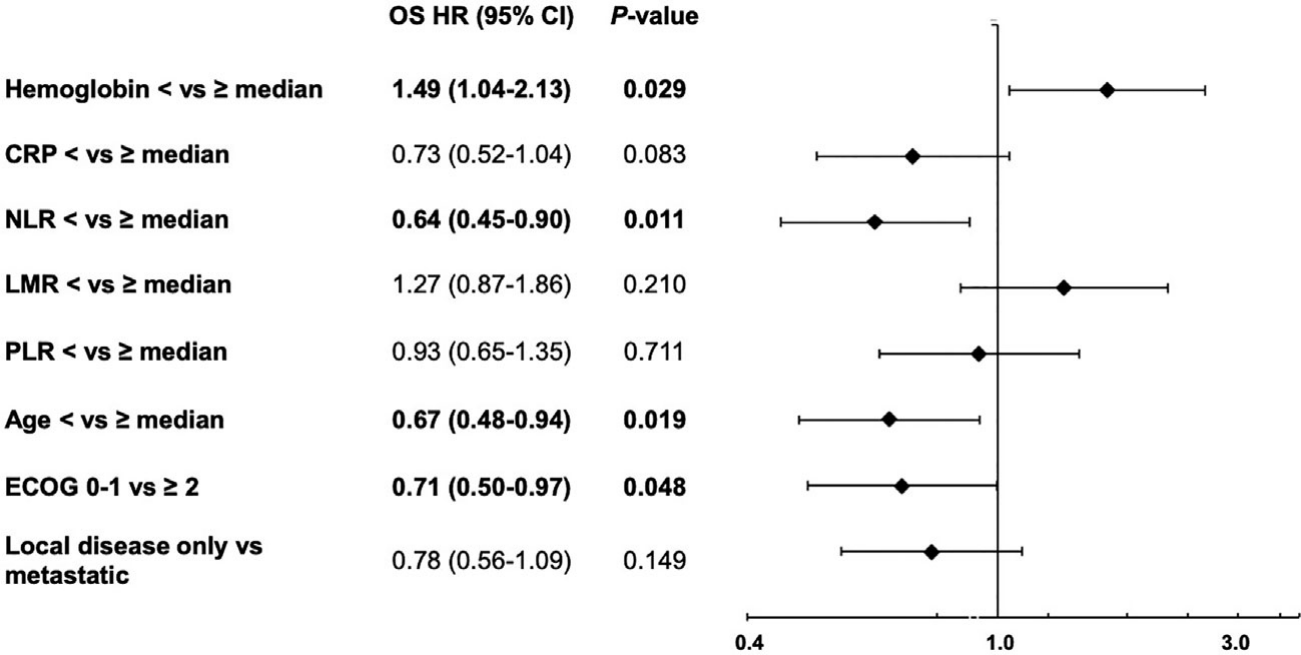
Forest plot for the multivariate analysis of overall survival from start of first-line therapy, including hemoglobin, C-reactive protein (CRP), neutrophil/lymphocyte ratio (NLR), lymphocyte/monocyte ratio (LMR), platelets/lymphocyte ratio (PLR), age, ECOG performance status and extend of disease (local disease only *vs* metastatic).

First, the impact of all eight parameters on OS were analyzed in exploratory univariate analyses (**Table 5**). Here, we identified anemia, CRP, NLR, LMR and ECOG as prognostic factors for overall survival. Afterwards, we performed a multivariate Cox proportional-hazard analysis including all eight parameters to verify the independence of the identified prognostic markers. In this multivariate analysis, only anemia, NLR, age and ECOG status significantly correlated with OS (*P* < 0.05) and were therefore considered as independent prognostic markers (**Figure 3**).

**Table 5:** Median overall survival from start of first-line therapy and hazard ratio (univariate analysis).

	Median overall survival (months)	*P-*value	*N*	Hazard ratio (with 95% confidence intervals)
Hemoglobin < *vs* ≥ median	9.89 *vs* 13.01	<0.001	210	1.72 (1.28–2.33, *P* < 0.001)
CRP < *vs* ≥ median	13.44 *vs* 9.13	0.001	205	0.61 (0.45–0.82, *P* = 0.001)
NLR < *vs* ≥ median	13.44 *vs* 8.30	0.007	192	0.66 (0.49–0.90, *P* = 0.008)
LMR < *vs* ≥ median	9.23 *vs* 12.58	0.023	192	1.41 (1.05–1.92, *P* = 0.024)
PLR < *vs* ≥ median	12.85 *vs* 8.54	0.151	192	0.80 (0.59–1.09, *P* = 0.152)
Age < *vs* ≥ median	10.58 *vs* 12.58	0.108	218	0.79 (0.60–1.05, *P* = 0.109)
ECOG 0–1 *vs* ≥2	12.19 *vs* 9.46	0.027	203	0.71 (0.52–0.96, *P* = 0.028)
Local disease only vs metastatic disease	11.50 *vs* 11.53	0.741	218	1.05 (0.79–1.39, *P* = 0.741)

NLR, neutrophil–lymphocyte ratio; LMR, lymphocyte–monocyte ratio; PLR, platelet–lymphocyte ratio.

### Prognostic Clinical Score

**Figure 4:**
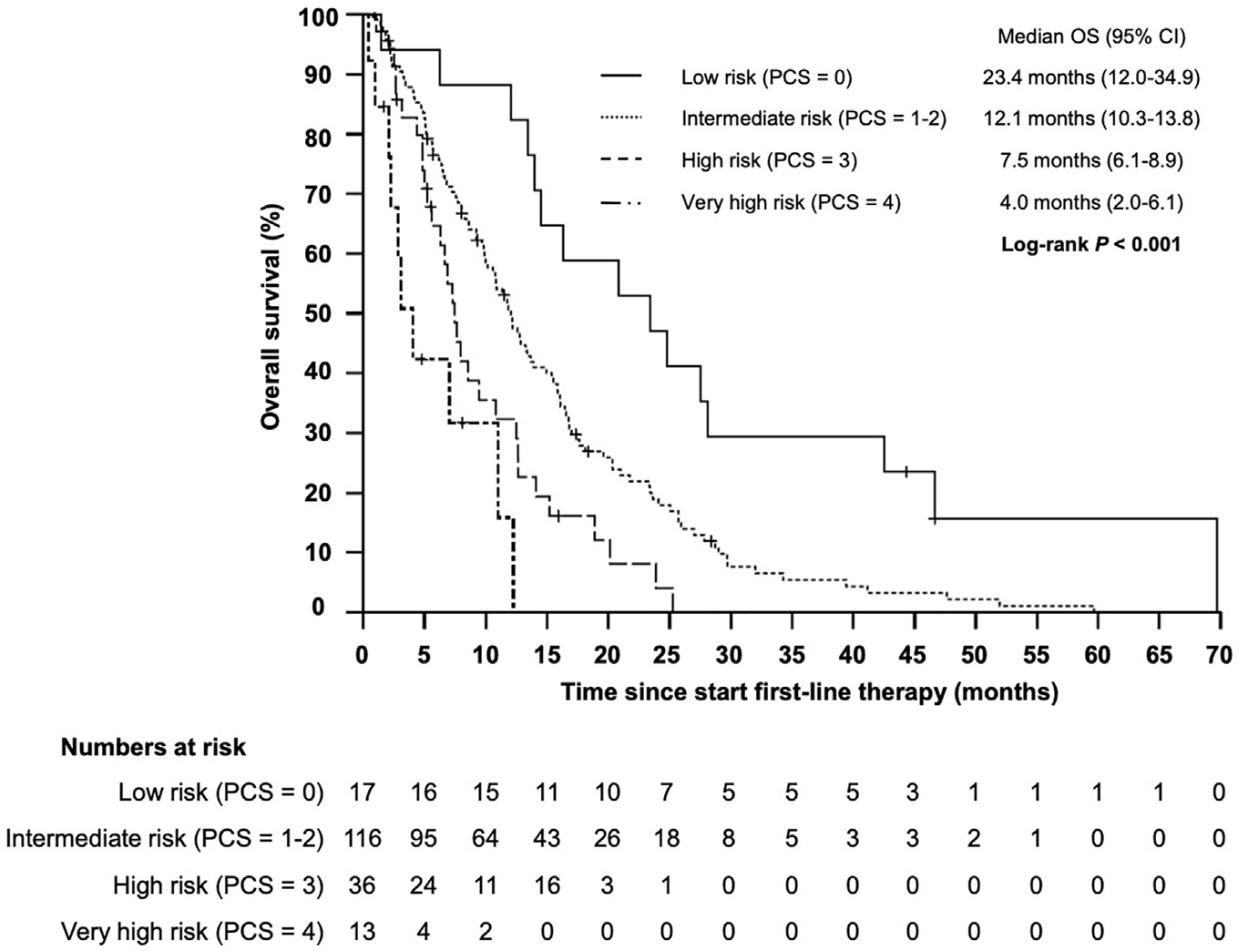
Kaplan–Meier plot for overall survival (OS) according to the prognostic clinical score (PCS) from start of first-line therapy for low (PCS = 0 points), intermediate (PCS = 1–2 points), high (PCS = 3 points), and very high risk (PCS = 4 points) groups (*P* < 0.001).

**Figure 5:**
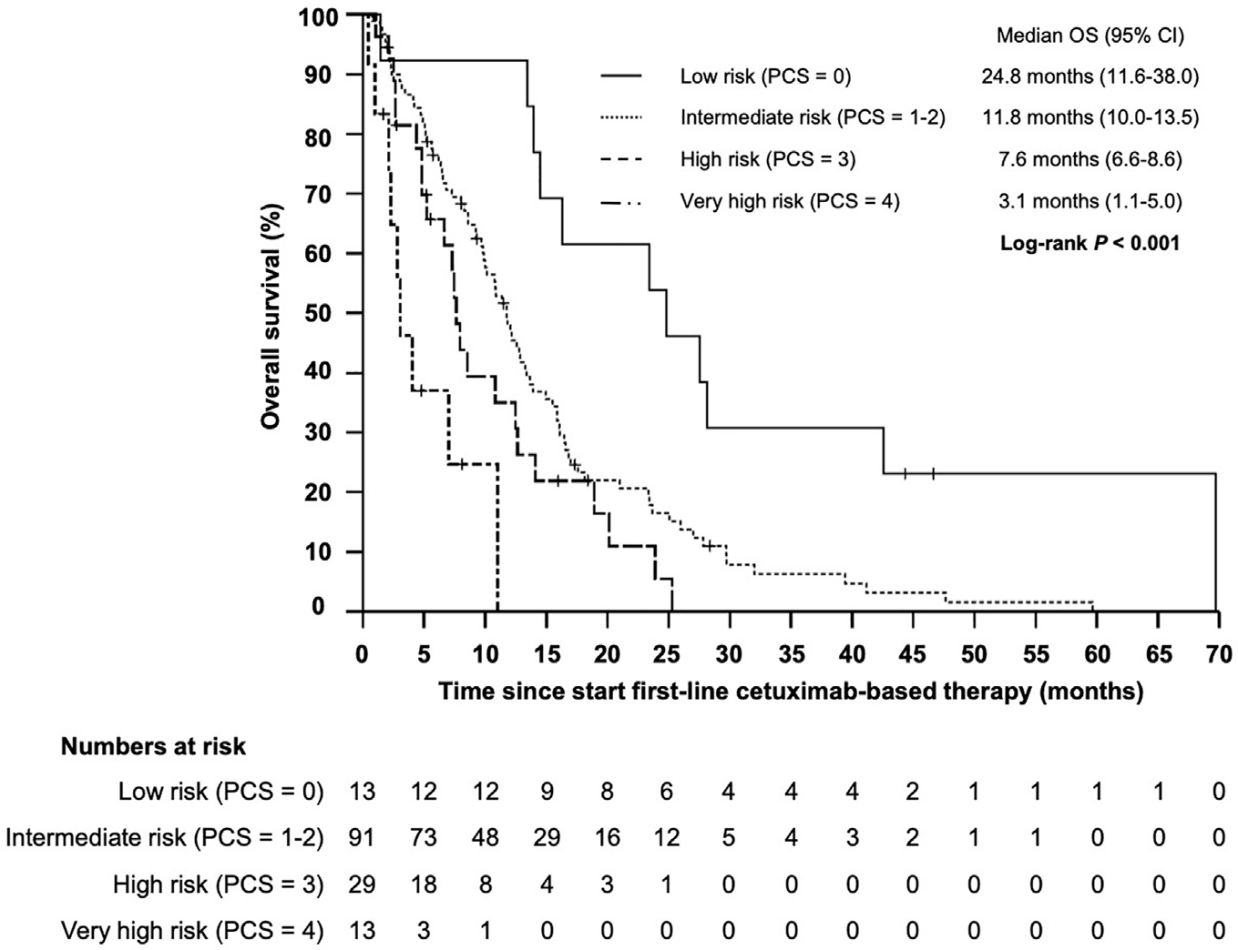
Kaplan–Meier plot for overall survival (OS) according to the prognostic clinical score (PCS) from start of cetuximab-based first-line therapy for low (PCS = 0 points), intermediate (PCS = 1–2 points), high (PCS = 3 points) and very high risk (PCS = 4 points) groups (*P* < 0.001).

Based on the four identified independent prognostic markers, we establish a prognostic model (prognostic clinical score—PCS) for survival upon chemoimmunotherapy including cetuximab for r/m SCCHN. Due to a comparable effect size for each marker, one point was allocated for each positive factor. A marker was considered positive if the following condition was met: NLR ≥5.73; hemoglobin <11.80; age ≥61.8; ECOG ≥2. Next, we stratified patients into four groups (0, 1–2, 3, and 4 points) and correlated the different groups with the OS (**Figure 4**). Thereby, we identified patients with a favorable prognosis (PCS = 0, median OS 23.4 months, 95% CI 12.0 - 34.9), an intermediate prognosis (PCS = 1–2, median OS 12.1 months, 95% CI 10.3–13.8), a poor prognosis (PCS = 3, median OS 7.5 months, 95% CI 6.1–8.9) and a very poor prognosis (PCS = 4, median OS 4.0 months, 95% CI 2.0–6.1 months), *P* < 0.001. Each risk group hat a statistically significant different prognosis: HR for low *vs* intermediate risk was 0.434 (95% CI 0.246–0.765; *P* = 0.004); HR for intermediate *vs* poor risk was 0.552 (95% CI 0.365–0.834; *P* = 0.005); and the HR for poor *vs* very poor risk was 0.471 (95% CI 0.219–0.998; *P* = 0.046). Next, to exclude potential bias, we evaluated the PCS in the homogenous subgroup of patients which received cetuximab in first line and for whom all clinical-and blood-based parameters where available (*N* = 146). In this subgroup the prognostic value of the PCS could be confirmed (**Figure 5**, *P* < 0.001).

### Impact of Clinical- and Blood-Based Parameters on PFS and ORR Upon Cetuximab

**Figure 6:**
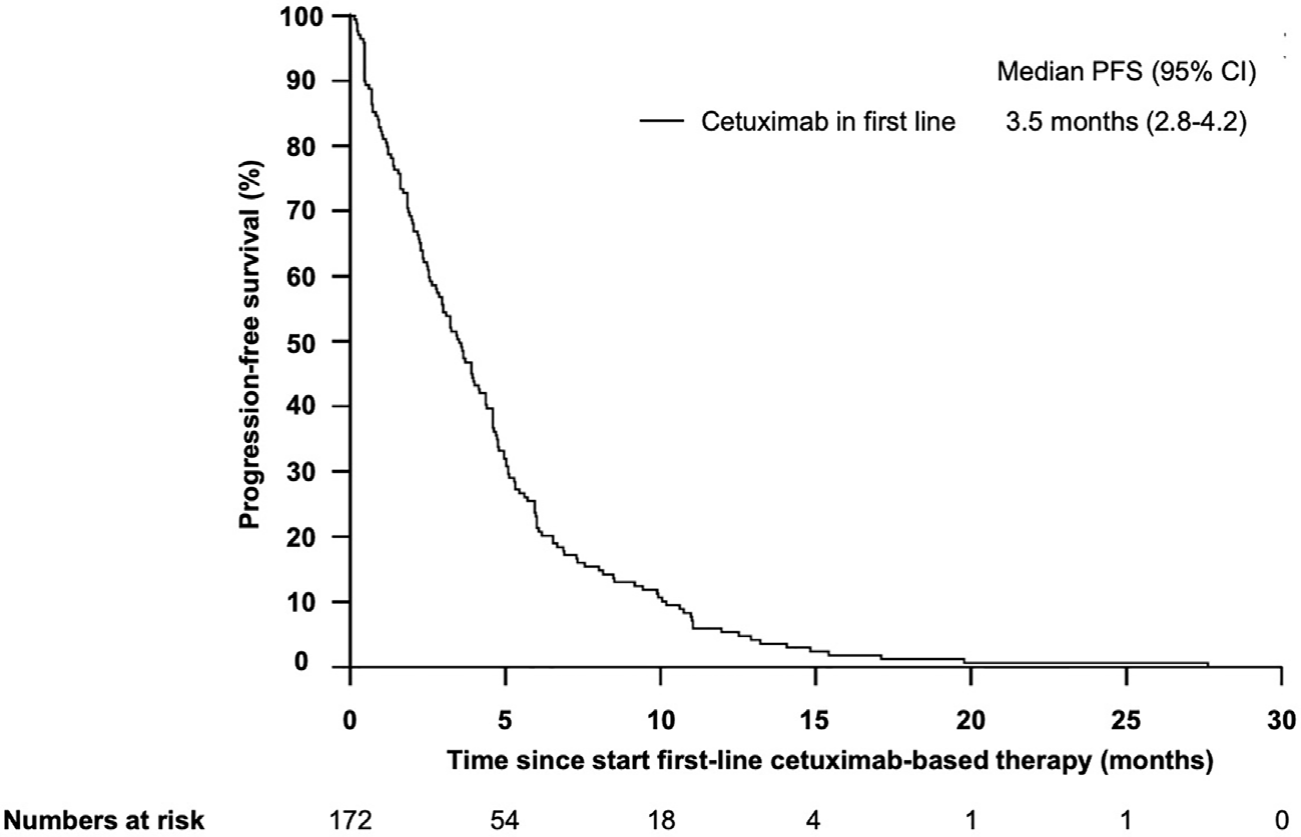
Kaplan–Meier plot for progression-free survival (PFS) for patients with r/m SCCHN from start of first-line cetuximab-based therapy. The median PFS was 3.5 months (95%CI 2.8–4.2).

**Figure 7:**
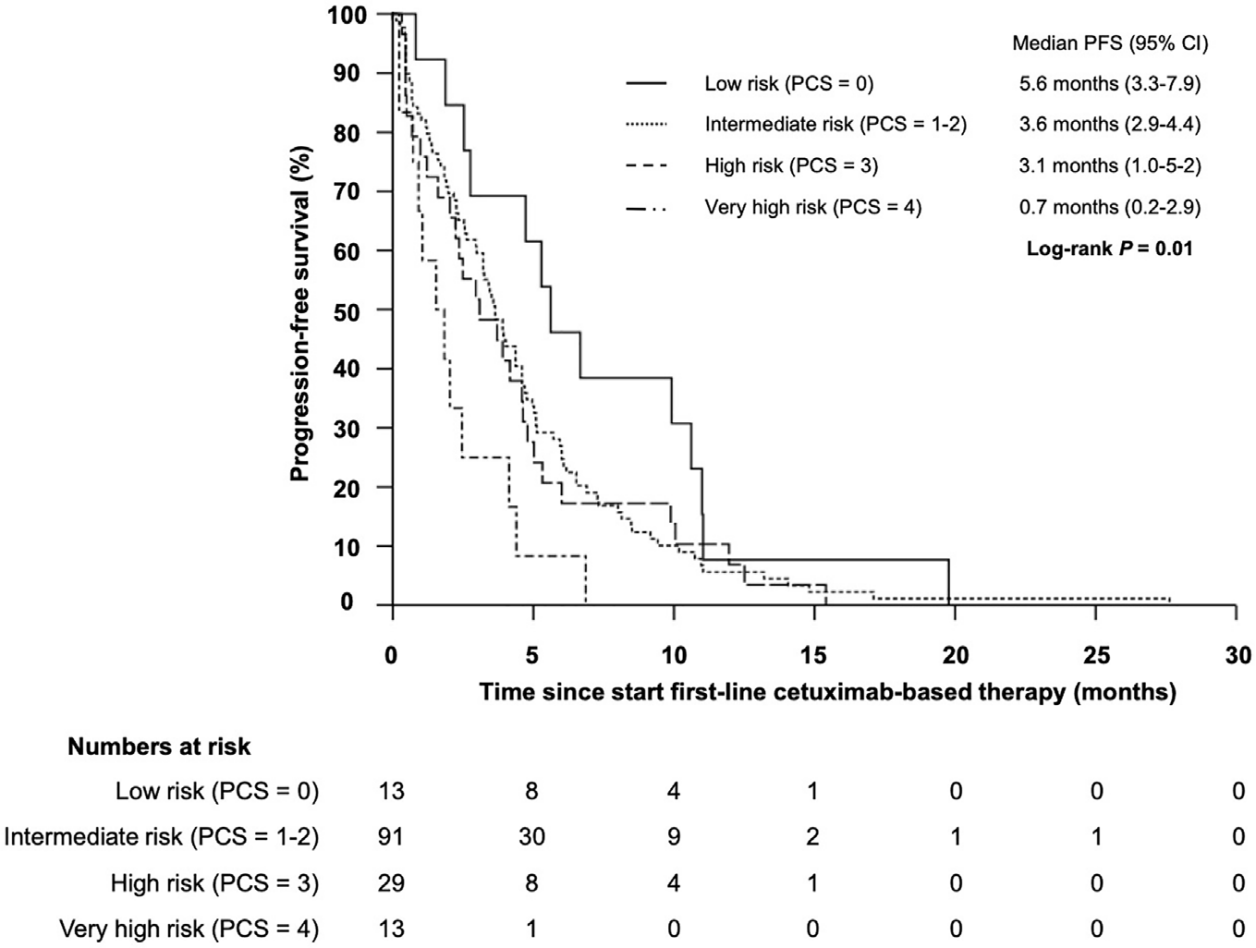
Kaplan–Meier plot for progression-free survival (PFS) for patients with r/m SCCHN from start of first-line cetuximab-based therapy according to the prognostic clinical score for low (PCS = 0 points), intermediate (PCS = 1–2 points), high (PCS = 3 points) and very high risk (PCS = 4 points) groups (log-rank P = 0.01).

**Figure 8:**
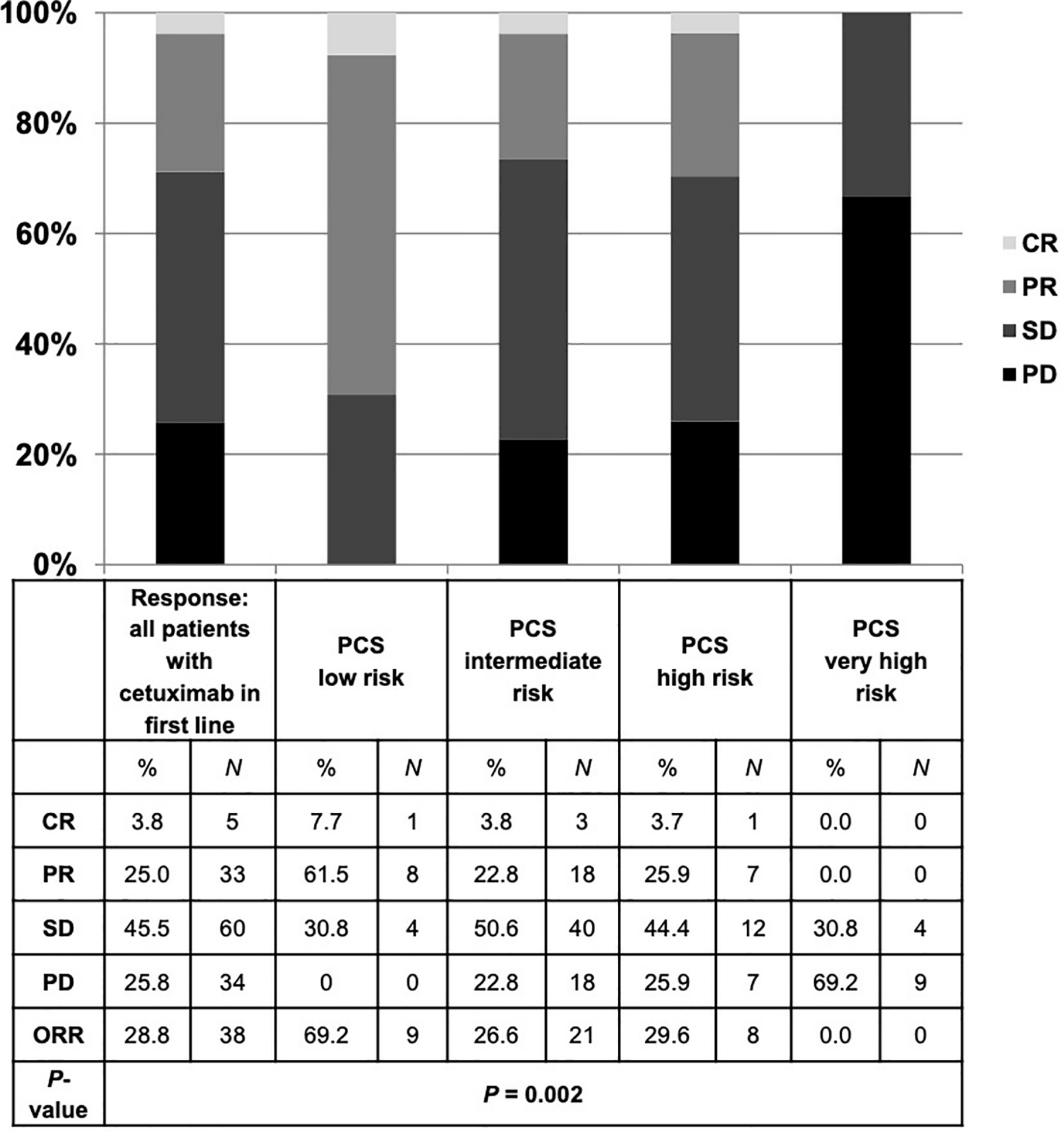
Best response to cetuximab-based first-line therapy for all patients and in each risk group of the prognostic clinical score (PCS). Patients with low risk PCS had a favorable overall response rate (ORR) of 69.2% and disease control rate (DCR) of 100%, whereas no objective response was detected in the very high risk group. CR, complete remission; PR, partial remission; SD, stable disease; PD, progressive disease.

The median progression-free survival (PFS) in patients who received cetuximab-based first-line therapy was 3.5 months (95% CI 2.8–4.2) (**Figure 6**). Comparable to the OS, median PFS differed significantly between patients with different PCS. Patients in the low risk group had a median PFS of 5.6 months, whereas patients in the very high risk group only had a median PFS of 0.7 months (**Figure 7**, *P* = 0.01). In line, patients with low risk PCS had a favorable overall response rate (ORR) of 69.2% and a disease control rate (DCR) of 100%, whereas no objective response was observed in the very high risk group (**Figure 8**).

### Impact of Cisplatin- or Carboplatin-Based First-Line Therapy

**Figure 9:**
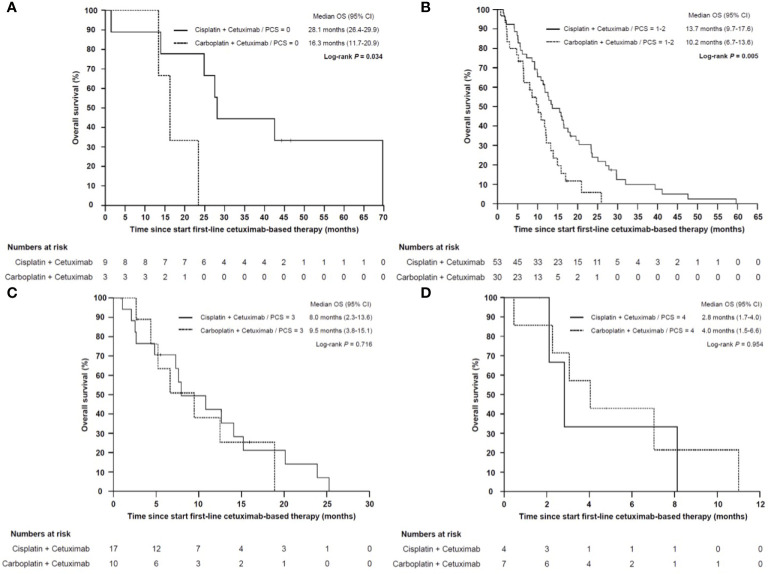
Kaplan–Meier plot for overall survival (OS) from start of cisplatin/cetuximab- *vs* carboplatin/cetuximab-based first-line therapy in low **(A)**, intermediate **(B)**, high **(C)** and very high risk **(D)** sub-groups. **(A)** Patients with low (PCS = 0) or **(B)** intermediate risk (PCS = 1–2) who were treated with cisplatin/cetuximab had a statistically significant prolonged overall survival compared to carboplatin/cetuximab (*P* = 0.034 and *P* = 0.005, respectively). **(C)** In high risk (PCS = 3) and **(D)** very high risk subgroups (PCS = 4) the prognosis remained poor regardless whether cisplatin or carboplatin was administered (*P* = 0.716 and *P* = 0.954, respectively).

To address the question, if the poor prognosis of patients with a high PCS could be overcome by a more intensive chemotherapy, we performed a subgroup analysis in patients which received cisplatin/cetuximab or carboplatin/cetuximab in first line. Patients who received cisplatin/cetuximab-based therapy had a significant prolonged OS compared to patients treated with carboplatin/cetuximab (13.7 months *vs* 9.5 months, HR 0.487, 95% CI 0.329–0.720, *P* < 0.001). However, only patients in the good and intermediate prognosis group (PCS 0-2) benefitted from the more intensive cisplatin-based therapy (28.1 *vs* 16.3 months for PCS 0, *P* = 0.034 and 13.7 *vs* 10.2 months for PCS 1-2, *P* = 0.005). In contrast, patients in the poor and very poor prognosis group had a dramatically shorter median OS irrespectively of cisplatin- or carboplatin-based first-line therapy (**Figure 9**).

## Discussion

Cetuximab-based chemotherapy has been the standard first-line therapy for patients with r/m SCCHN for the last decade and it is still a standard option for patients who progressed on or are not candidates for immunotherapy with checkpoint inhibitors (1). However, prognostic or predictive biomarkers for patients treated with cetuximab are still missing. In this study, we aimed to identify clinical and routinely assessed blood-based and clinical biomarkers for the efficacy of cetuximab-based chemoimmunotherapies in recurrent or metastatic disease. We focused on markers on inflammation and immune response, which are two essential hallmarks of cancer (3, 4, 16, 17). In our retrospective study, 218 patients with r/m SCCHN were included who received at least one cycle of palliative chemotherapy in combination with cetuximab, irrespectively of ECOG performance status, comorbidities, age, abnormal blood values or previous therapy. The median OS in our unselected patient population was 11.5 months which were highly comparable with the results of the pivotal EXTREME trial (median OS 10.1 months) or the patients population treated with the EXTREME regimen in the recently published KEYNOTE-048 study (median OS 10.7 months) (1, 4). Although our cohort represents a real-world population with nearly 20% of patients being older than 70 years and one third of patients with an ECOG performance status ≥2, the median OS was markedly longer than those reported in comparable retrospective studies like the GLANCE H&N (5). This finding could possibly be explained by the relative high number of patients receiving second and further lines therapies at our center.

Absolute neutrophil, monocyte and platelet counts, CRP, and anemia are easily accessible blood-based biomarkers to assess the systemic inflammation. In addition, lower lymphocyte count may be associated with immunodeficiency and could have an impact on the efficacy of immunomodulating agents like the monoclonal antibody cetuximab, which has the capability to mediate antibody dependent cellular cytotoxicity (ADCC) (18). By using ratios of these different blood parameters like the neutrophil–lymphocyte ratio (NLR) or the lymphocyte–monocyte ratio (LMR) the extent of the systemic inflammation and the competence of the immune system could be easily assessed. The NLR has been previously linked to a higher risk of recurrence after curative resection in locally SCCHN (19). Moreover, it has been described as a prognostic marker for a wide variety of other malignancies treated with palliative immunotherapy or chemotherapy (12, 13). In our patient population, a higher NLR was an independent prognostic marker for OS in the recurrent or metastatic disease upon palliative treatment. Here, we chose the median NLR of 5.73 as a cut-off. In previous published studies in patients with early and advanced stage SCCHN, cut-offs between 1.29 and 6.0 have been proposed (20). We additionally tested NLR cut-offs of 4, 5 and 6, which all discriminated patients with good and poor prognosis (*data not shown*). This is in line with a large meta-analysis published by Cho et al., 2018 (20). Thus, the NLR seems to be a continues instead of a simple dichotomous biomarker (21).

Cancer-related anemia is common in a wide variety of malignancies. Up to 60% of SCCHN patients present with reduced hemoglobin levels at initial diagnosis (22). Cytokine-mediated systemic inflammation with increased interleukin-1 and -6 levels and nutritional and metabolic alterations contribute to this multifactorial paraneoplastic syndrome (23). The prognostic significance of pre-treatment cancer related anemia has been demonstrated for curative treated SCCHN patients (9, 24, 25). In our cohort of palliative treated r/m SCCHN patients, we chose the median hemoglobin level (11.8 mg/dl) as cut-off for further analyses. We could clearly demonstrate that anemia was independently associated with a reduced OS in our cohort. This is in contrast to a previous study published by Magnes et al. in a smaller cohort of r/m SCCHN patients (26). However, a different cut-off and heterogeneous patient’s characteristics or therapeutic strategies could have affected the results.

Recently, pembrolizumab has been approved as monotherapy and in combination with platinum-based chemotherapy in first-line treatment of PD-L1 positive r/m SCCHN (4). Moreover, the TPEx protocol with cisplatin, docetaxel and cetuximab has emerged as a highly effective and less toxic regiment than the EXTREME protocol (27). These new options result in a broader therapeutic armamentarium in the first-line setting. However, predictive or prognostic biomarkers for the first-line treatment in r/m SCCHN are still missing and are urgently needed to identify the best therapeutic option for each patient. To this end, we developed an easy-to-use prognostic clinical scoring (PCS) system based on our identified independent clinical- and blood-based biomarkers. In the EXTREME trial, sub-group analysis suggested a benefit in median OS only for cetuximab in combination with cisplatin, but not for the combination with carboplatin (1). In line, we found a significant higher OS in patients treated with cisplatin/cetuximab compared to carboplatin/cetuximab in our real-world cohort. Interestingly, only patients in our new established good and intermediate PCS benefited from a cisplatin/cetuximab-based therapy compared to carboplatin/cetuximab. In contrast, patients with a poor (PCS = 3) and very poor prognosis (PCS = 4) had a dramatic reduced OS irrespectively of the type of first-line therapy.

Our analysis has some important limitations. The data were collected retrospectively and all patients were treated at a single institution. The decision of first- and further line therapies were made individually by the clinicians and/or patients and not according to a pre-specified protocol. This real-world scenario resulted in a more heterogeneous treatment selection with cetuximab given not always in first-line and not always in combination with platin/5-fluorouracil. We adapted to this specific limitation by testing the established PCS in the subset of patients who received cetuximab in the first-line setting.

In conclusion, we identified age, ECOG, NLR and anemia as independent prognostic factors for patients with r/m SCCHN treated with cetuximab-based therapy. By combining these four independent parameters we established a new prognostic clinical score (PCS) which could discriminate patients in groups with good (PCS = 0), intermediate (PCS = 1–2), poor (PCS = 3) and very poor (PCS = 4) prognosis. With the PCS we characterize a subgroup of patients who were most likely to respond to cetuximab-based first-line therapy, and a subgroup of patients without any response to this chemoimmunotherapy in the first-line setting. Furthermore, the PCS enables to distinguish between patients who benefit from an intensive cisplatin/cetuximab-based first-line therapy (patients with good and intermediate PCS) and those patients with poor outcome irrespectively of the selected first-line therapy (patients with poor and very poor PCS). Based on this findings, the PCS will help to guide treatment selection for patients with PD-L1 negative r/m SCCHN, patients with contraindications for CPI and patients who progressed on CPI. Cisplatin/cetuximab-based therapy should be considered as the standard first-line option for patients with a PCS of 0 or 1–2, for patients with a PCS of 3 carboplatin/cetuximab is an adequate option due to a better toxicity profile and comparable efficacy in this subgroup, and best supportive care should be considered in patients with a PCS of 4.

As a next step, we plan an external and subsequently prospective validation of the PCS in an independent patient cohort. Finally, the new proposed PCS may help in the design of risk-adapted treatment strategies in future clinical trials.

## Data Availability Statement

The datasets generated for this study are available on request to the corresponding author.

## Ethics Statement

The studies involving human participants were reviewed and approved by Local Ethics Committee of the Medical Faculty of the University Duisburg-Essen (Az 13-5486-BO). Written informed consent for participation was not required for this study in accordance with the national legislation and the institutional requirements.

## Author Contributions

SK and MP were responsible for conception and design of this study. TH, MP, SK, TG, MSt, ST, BK, KS, SL, VG, and MSc collected the data. MP, TH, and SK performed the data extraction and statistical analyses. MP and SK wrote the manuscript. All authors contributed to the article and approved the submitted version.

## Funding

This project was supported by a grant of the “Stiftung Tumorforschung Kopf-Hals” to SK. The West German Cancer Center at University Hospital Essen is supported by the Oncology Center of Excellence Program of the German Cancer Aid (grant number 110534), and the German Federal and North Rhine-Westphalian State governments as partner site of the German Cancer Consortium (DKTK).

## Conflict of Interest

MSc: Consultancy: AstraZeneca, Boehringer Ingelheim, Bristol-Myers Squibb, Celgene, Institut für Qualität und Wirtschaftlichkeit im Gesundheitswesen (IQWiG), Lilly, Novartis; Honoraria for CME presentations: Alexion, Boehringer Ingelheim, Celgene, GlaxoSmithKline, Lilly, Novartis; Research funding to institution: Boehringer Ingelheim, Bristol Myers-Squibb, Novartis; Other: Universität Duisburg-Essen (Patents). SK: Consultancy, Honoraria and Travel Support: Roche, Bristol-Myers Squibb, Merck Sharp & Dohme, Amgen, Merck Serono, Sanofi Aventis, Astra Zeneca, Janssen Pharmaceuticals, Celgene, Lilly, Servier. MP: Consultancy, Honoraria and Travel Support: Boehringer Ingelheim, Bristol Myers-Squibb, Merck Healthcare KGaA, Merck Sharp & Dohme. TG: Consultancy, Honoraria and Travel Support: Astra Zeneca, Bristol Myers-Squibb, Merck Healthcare KGaA, Merck Sharp & Dohme, Roche. VG: grants, personal fees and non-financial support from Astra Zeneca, Bristol-Myers Squibb, Ipsen, Pfizer; grants and personal fees from Novartis, personal fees from Eisai, EUSAPharm, Roche, Janssen-Cilag, Merck Serono, MSD Sharp & Dohme, Lilly, PharmaMar, Asklepios Clinic, Clinic of Oldenburg, Diakonie Clinic, Dortmund Hospital, and Onkowissen.

The remaining authors declare that the research was conducted in the absence of any commercial or financial relationships that could be construed as a potential conflict of interest.

## References

[1] Vermorken JB Mesia R Rivera F Remenar E Kawecki A Rottey S. Platinum-Based Chemotherapy Plus Cetuximab in Head and Neck Cancer. N Engl J Med (2008) 359(11):1116–27. 10.1056/NEJMoa0802656 18784101

[2] Taberna M Oliva M Mesia R. Cetuximab-Containing Combinations in Locally Advanced and Recurrent or Metastatic Head and Neck Squamous Cell Carcinoma. Front Oncol (2019) 9:383. 10.3389/fonc.2019.00383 31165040 PMC6536039

[3] Ferris RL Blumenschein G Jr Fayette J Guigay J Colevas AD Licitra L. Nivolumab for Recurrent Squamous-Cell Carcinoma of the Head and Neck. N Engl J Med (2016) 375(19):1856–67. 10.1056/NEJMoa1602252 PMC5564292 27718784

[4] Burtness B Harrington KJ Greil R Soulieres D Tahara M de Castro G Jr.. Pembrolizumab Alone or With Chemotherapy Versus Cetuximab With Chemotherapy for Recurrent or Metastatic Squamous Cell Carcinoma of the Head and Neck (KEYNOTE-048): A Randomised, Open-Label, Phase 3 Study. Lancet (2019) 394(10212):1915–28. 10.1016/S0140-6736(19)32591-7 31679945

[5] Grunwald V Chirovsky D Cheung WY Bertolini F Ahn MJ Yang MH. Global Treatment Patterns and Outcomes Among Patients With Recurrent and/or Metastatic Head and Neck Squamous Cell Carcinoma: Results of the GLANCE H&N Study. Oral Oncol (2020) 102:104526. 10.1016/j.oraloncology.2019.104526 31978755

[6] Galot R Le Tourneau C Guigay J Licitra L Tinhofer I Kong A. Personalized Biomarker-Based Treatment Strategy for Patients With Squamous Cell Carcinoma of the Head and Neck: EORTC Position and Approach. Ann Oncol (2018) 29(12):2313–27. 10.1093/annonc/mdy452 30307465

[7] Polee MB Hop WC Kok TC Eskens FA van der Burg ME Splinter TA. Prognostic Factors for Survival in Patients With Advanced Oesophageal Cancer Treated With Cisplatin-Based Combination Chemotherapy. Br J Cancer (2003) 89(11):2045–50. 10.1038/sj.bjc.6601364 PMC2376851 14647136

[8] Jang RW Caraiscos VB Swami N Banerjee S Mak E Kaya E. Simple Prognostic Model for Patients With Advanced Cancer Based on Performance Status. J Oncol Pract (2014) 10(5):e335–41. 10.1200/JOP.2014.001457 25118208

[9] Fakhry C Zhang Q Nguyen-Tan PF Rosenthal DI Weber RS Lambert L. Development and Validation of Nomograms Predictive of Overall and Progression-Free Survival in Patients With Oropharyngeal Cancer. J Clin Oncol (2017) 35(36):4057–65. 10.1200/JCO.2016.72.0748 PMC5736236 28777690

[10] Cummings M Merone L Keeble C Burland L Grzelinski M Sutton K. Preoperative Neutrophil:Lymphocyte and Platelet:Lymphocyte Ratios Predict Endometrial Cancer Survival. Br J Cancer (2015) 113(2):311–20. 10.1038/bjc.2015.200 PMC4506386 26079303

[11] Proctor MJ Morrison DS Talwar D Balmer SM Fletcher CD O’Reilly DS. A Comparison of Inflammation-Based Prognostic Scores in Patients With Cancer. A Glasgow Inflammation Outcome Study. Eur J Cancer (2011) 47(17):2633–41. 10.1016/j.ejca.2011.03.028 21724383

[12] Bilen MA Martini DJ Liu Y Lewis C Collins HH Shabto JM. The Prognostic and Predictive Impact of Inflammatory Biomarkers in Patients Who Have Advanced-Stage Cancer Treated With Immunotherapy. Cancer (2019) 125(1):127–34. 10.1002/cncr.31778 30329148

[13] Chua W Charles KA Baracos VE Clarke SJ. Neutrophil/Lymphocyte Ratio Predicts Chemotherapy Outcomes in Patients With Advanced Colorectal Cancer. Br J Cancer (2011) 104(8):1288–95. 10.1038/bjc.2011.100 PMC3078587 21448173

[14] Therasse P Arbuck SG Eisenhauer EA Wanders J Kaplan RS Rubinstein L. New Guidelines to Evaluate the Response to Treatment in Solid Tumors. European Organization for Research and Treatment of Cancer, National Cancer Institute of the United States, National Cancer Institute of Canada. J Natl Cancer Inst (2000) 92(3):205–16. 10.1093/jnci/92.3.205 10655437

[15] Eisenhauer EA Therasse P Bogaerts J Schwartz LH Sargent D Ford R. New Response Evaluation Criteria in Solid Tumours: Revised RECIST Guideline (Version 1.1). Eur J Cancer (2009) 45(2):228–47. 10.1016/j.ejca.2008.10.026 19097774

[16] Balkwill F Mantovani A. Inflammation and Cancer: Back to Virchow? Lancet (2001) 357(9255):539–45. 10.1016/S0140-6736(00)04046-0 11229684

[17] Hanahan D Weinberg RA. Hallmarks of Cancer: The Next Generation. Cell (2011) 144(5):646–74. 10.1016/j.cell.2011.02.013 21376230

[18] Kasper S Breitenbuecher F Reis H Brandau S Worm K Kohler J. Oncogenic RAS Simultaneously Protects Against anti-EGFR Antibody-Dependent Cellular Cytotoxicity and EGFR Signaling Blockade. Oncogene (2013) 32(23):2873–81. 10.1038/onc.2012.302 22797062

[19] Rassouli A Saliba J Castano R Hier M Zeitouni AG. Systemic Inflammatory Markers as Independent Prognosticators of Head and Neck Squamous Cell Carcinoma. Head Neck (2015) 37(1):103–10. 10.1002/hed.23567 24339165

[20] Cho JK Kim MW Choi IS Moon UY Kim MJ Sohn I. Optimal Cutoff of Pretreatment Neutrophil-to-Lymphocyte Ratio in Head and Neck Cancer Patients: A Meta-Analysis and Validation Study. BMC Cancer (2018) 18(1):969. 10.1186/s12885-018-4876-6 30309318 PMC6182814

[21] Lecot P Sarabi M Pereira Abrantes M Mussard J Koenderman L Caux C. Neutrophil Heterogeneity in Cancer: From Biology to Therapies. Front Immunol (2019) 10:2155. 10.3389/fimmu.2019.02155 31616408 PMC6764113

[22] Maccio A Madeddu C Gramignano G Mulas C Tanca L Cherchi MC. The Role of Inflammation, Iron, and Nutritional Status in Cancer-Related Anemia: Results of a Large, Prospective, Observational Study. Haematologica (2015) 100(1):124–32. 10.3324/haematol.2014.112813 PMC4281325 25239265

[23] Madeddu C Gramignano G Astara G Demontis R Sanna E Atzeni V. Pathogenesis and Treatment Options of Cancer Related Anemia: Perspective for a Targeted Mechanism-Based Approach. Front Physiol (2018) 9:1294. 10.3389/fphys.2018.01294 30294279 PMC6159745

[24] Baumeister P Canis M Reiter M. Preoperative Anemia and Perioperative Blood Transfusion in Head and Neck Squamous Cell Carcinoma. PLoS One (2018) 13(10):e0205712. 10.1371/journal.pone.0205712 30347001 PMC6197687

[25] Dubray B Mosseri V Brunin F Jaulerry C Poncet P Rodriguez J. Anemia is Associated With Lower Local-Regional Control and Survival After Radiation Therapy for Head and Neck Cancer: A Prospective Study. Radiology (1996) 201(2):553–8. 10.1148/radiology.201.2.8888257 8888257

[26] Magnes T Melchardt T Weiss L Mittermair C Neureiter D Klieser E. Prognostic Score in Patients With Recurrent or Metastatic Carcinoma of the Head and Neck Treated With Cetuximab and Chemotherapy. PLoS One (2017) 12(7):e0180995. 10.1371/journal.pone.0180995PMCPMC5501656 28686697 PMC5501656

[27] Guigay J Aupérin A Fayette J Saada-Bouzid E Lafond C Taberna M. Cetuximab, Docetaxel, and Cisplatin Versus Platinum, Fluorouracil, and Cetuximab as First-Line Treatment in Patients With Recurrent or Metastatic Head and Neck Squamous-Cell Carcinoma (GORTEC 2014-01 TPExtreme): A Multicentre, Open-Label, Randomised, Phase 2 Trial. Lancet Oncol (2021) S1470-2045(20):30755–5. 10.1016/S1470-2045(20)30755-5 33684370

