# Supplementary material for: Identification of a Prognostic Clinical Score for Patients With Recurrent or Metastatic Squamous Cell Carcinoma of the Head and Neck Treated With Systemic Therapy Including Cetuximab
Source: Front Oncol. 2021 May 13;11:635096. doi: 10.3389/fonc.2021.635096 (PMC8155676; doi:10.3389/fonc.2021.635096)
Supplement: Supplementary file 1 [file Presentation_1.pptx]

## Slide 1
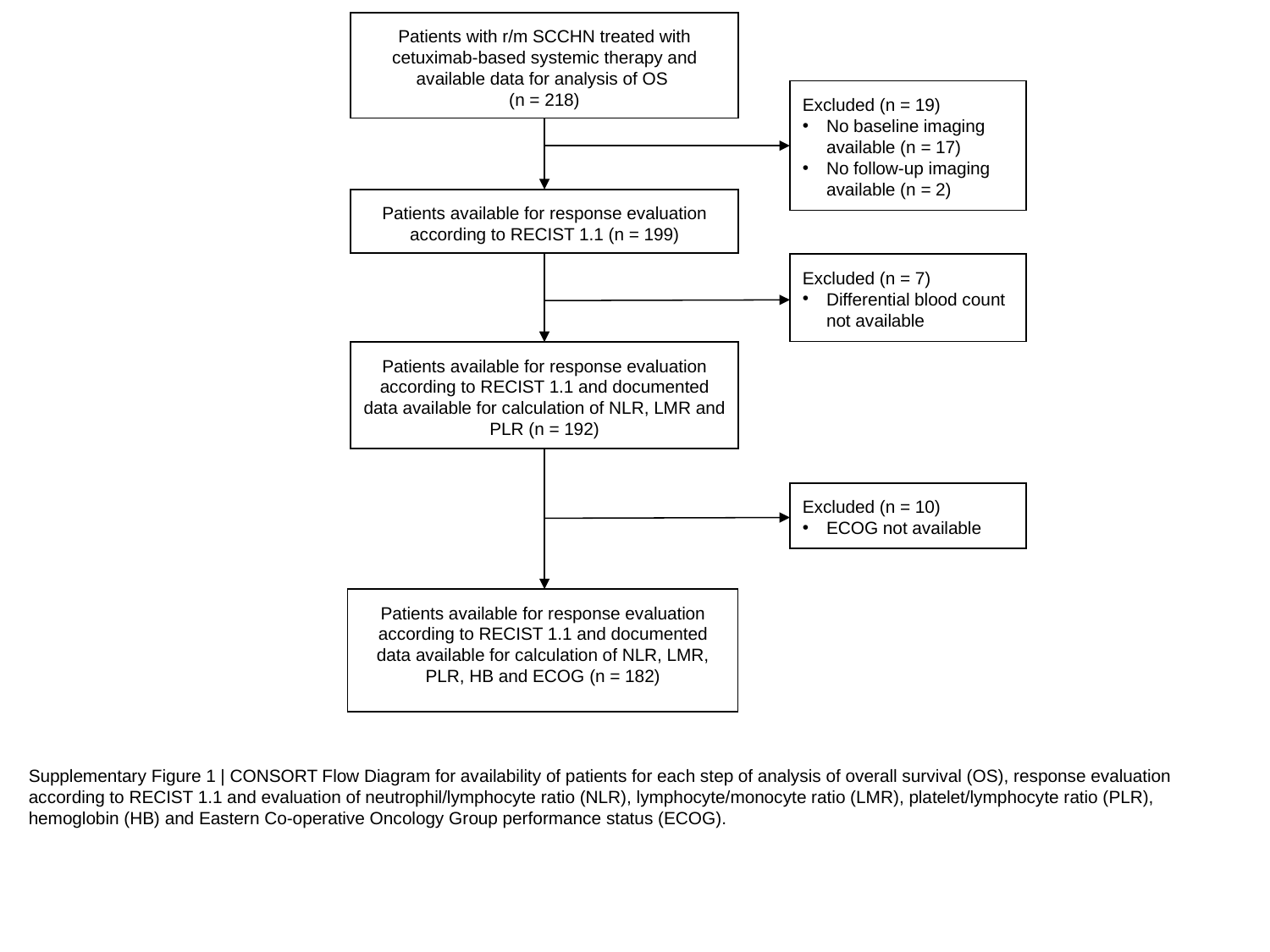

Patients with r/m SCCHN treated with cetuximab-based systemic therapy and available data for analysis of OS
(n = 218)
Excluded (n = 19)
No baseline imaging available (n = 17)
No follow-up imaging available (n = 2)
Patients available for response evaluation according to RECIST 1.1 (n = 199)
Excluded (n = 7)
Differential blood count not available
Patients available for response evaluation according to RECIST 1.1 and documented data available for calculation of NLR, LMR and PLR (n = 192)
Excluded (n = 10)
ECOG not available
Patients available for response evaluation according to RECIST 1.1 and documented data available for calculation of NLR, LMR, PLR, HB and ECOG (n = 182)
Supplementary Figure 1 | CONSORT Flow Diagram for availability of patients for each step of analysis of overall survival (OS), response evaluation according to RECIST 1.1 and evaluation of neutrophil/lymphocyte ratio (NLR), lymphocyte/monocyte ratio (LMR), platelet/lymphocyte ratio (PLR), hemoglobin (HB) and Eastern Co-operative Oncology Group performance status (ECOG).
